# Effects of scanning sensitivity and multiple scan algorithms on microarray data quality

**DOI:** 10.1186/1471-2105-11-127

**Published:** 2010-03-12

**Authors:** Andrew Williams, Errol M Thomson

**Affiliations:** 1 Population Health Studies Division, Environmental Health Science and Research Bureau, Health Canada, Ottawa, K1A 0K9, Canada; 2 Hazard Identification Division, Environmental Health Science and Research Bureau, Health Canada, Ottawa, K1A 0K9, Canada

## Abstract

**Background:**

Maximizing the utility of DNA microarray data requires optimization of data acquisition through selection of an appropriate scanner setting. To increase the amount of useable data, several approaches have been proposed that incorporate multiple scans at different sensitivities to reduce the quantification error and to minimize effects of saturation. However, no direct comparison of their efficacy has been made. In the present study we compared individual scans at low, medium and high sensitivity with three methods for combining data from multiple scans (either 2-scan or 3-scan cases) using an actual dataset comprising 40 technical replicates of a reference RNA standard.

**Results:**

Of the individual scans, the low scan exhibited the lowest background signal, the highest signal-to-noise ratio, and equivalent reproducibility to the medium and high scans. Most multiple scan approaches increased the range of probe intensities compared to the individual scans, but did not increase the dynamic range (the proportion of useable data). Approaches displayed striking differences in the background signal and signal-to-noise ratio. However, increased probe intensity range and improved signal-to-noise ratios did not necessarily correlate with improved reproducibility. Importantly, for one multiple scan method that combined 3 scans, reproducibility was significantly improved relative to individual scans and all other multiple scan approaches. The same method using 2 scans yielded significantly lower reproducibility, attributable to a lack-of-fit of the statistical model.

**Conclusions:**

Our data indicate that implementation of a suitable multiple scan approach can improve reproducibility, but that model validation is critical to ensure accurate estimates of probe intensity.

## Background

DNA microarrays allow analysis of genome-wide gene expression. While an entire transcriptome can theoretically be quantified on a single array, in practice a proportion of probes analysed will not provide quantifiable signal. For example, when scanning any complex biological sample hybridized to a microarray, low copy number genes may emit low fluorescence signals not detectable above background; conversely, high copy number genes may emit fluorescence signals that are saturated. To maximize the amount of data acquired from a single microarray scan, the user attempts to generate a scan that spans the entire intensity range by selecting appropriate photo-multiplier tube (PMT) settings. In selecting the PMT of the scanner, the user has two major concerns: quantification error associated with image analysis (e.g., distinguishing signal from background) and signal saturation associated with the selection of scanner sensitivity [1-12]. If the microarray is scanned at a low sensitivity, more probes may be within background and thus there is uncertainty associated with the measured signal intensity (quantification error). If the sensitivity is set too high, more probes will be saturated (data censoring). If filtering absent or saturated probes prior to statistical analysis, having probes in the background or probes with saturated signal intensities results in fewer probes that can be assessed for statistically significant changes.

The range of fluorescence intensities on an array is unknown prior to scanning. As a result, the optimal PMT setting is not known. Currently there is no objective guideline for selecting the optimum scanner setting [2,10,11]. However, a number of approaches have been proposed to maximize the amount of data acquired from an array. Using lower sensitivity scans to minimize the number of probes with saturated signal intensity may be considered preferable, as it ensures that data can be obtained for the greatest number of probes [3]. Others suggest that high sensitivity settings should be used to obtain quantifiable fluorescent signals from low copy number genes that may be close to background [2]. A third possibility is that if a small set of scans is available, the optimal scan may be determined following assessment by a given metric, such as the signal-to-noise ratio (SNR) [1,4]. As no single scan may be optimal for all probes, taking the union of data generated from a number of individual scans may be an effective method for extracting additional information from an array [11]. Having the majority of the expression data in the centre of the intensity range (i.e. around 256 fluorescent units) is an alternative metric [5,13]. However, Sharov et al. [12] suggested that this may result in too many probes within background, and so aiming for an average intensity [log(R × G)/2] between 10 to 12 (1024 to 4096 fluorescent units (FLUs)) may be preferable. Unless scanning at very high PMT, overall there are more probes in the background than at saturation. Approaches that improve quantification of low copy number probes would therefore be advantageous.

An appealing alternative to these single scan approaches is to combine data from two or more scans at different scanner sensitivities to yield an improved estimate of gene expression for a greater proportion of microarray probes [1-10]. Such an approach would use information acquired from both high and low sensitivity scans to improve quantification of low and high copy number genes respectively. This should theoretically increase the quantity of useable data. A number of multiple scan approaches have been presented in the literature [1-10]. To our knowledge, there has been no systematic comparison of multiple scan approaches. Multiple scan approaches do come at a cost, namely increased scanning and computational time compared with single scan approaches. Such a comparison would therefore be of use in determining whether a multiple scan approach is warranted, and if so, which multiple scan approach to use. In order to determine the best approach, a number of questions should be addressed: (1) Is reproducibility greater at low or high scanning intensity? (2) Do multiple scan approaches increase the dynamic range (i.e. the range of useable data above background and below 10% of saturation)? (3) Do combinations of scans of the same microarray improve data reproducibility? (4) Are there differences in the reproducibility of data among multiple scan approaches?

In the present study we used data produced in our laboratory from 40 technical replicates scanned sequentially at different laser settings to compare three different approaches to combining multiple scans [1-3]. The first approach (Lyng et al. [1]) estimates the multiplicative scaling effect of the scanner using a subset of the high intensity probes not affected by saturation. This scaling is then applied to the data obtained from the low scan to impute probes affected by saturation. The second approach (Garcia de la Nava et al. [2]) uses all the data to estimate the multiplicative scaling effect using robust regression and then minimizes an objective function for each probe to estimate the signal intensity. The third approach (Khondoker et al. [3]) uses a functional regression model to fit the data from multiple scans to obtain a single estimate of gene expression. Individual scans at low, medium and high sensitivity were compared with these multiple scan approaches using a variety of metrics including SNR, mean and coefficient of variation of the negative control probes, and proportion of useable data. To compare data reproducibility across approaches, a Spearman correlation analysis of the technical replicates was conducted. Our data indicate that employing a multiple scan approach can improve reproducibility over a subset of the intensity range.

## Results

To enable comparison of single scan and multiple scan approaches, we employed data from a recent 2-colour experiment [14] that included forty replicates of a commercially-available reference RNA. Mouse Universal Reference RNA (Stratagene, CA, USA) was labeled with Cy3 and hybridized to Agilent 22K DNA microarrays. Cy5-labelled lung RNA samples were also hybridized, as described previously [14], but data from the Cy5 channel were not used in the present analysis. Chips were scanned at 3 PMT settings on a ScanArray Express (Perkin-Elmer) scanner ranging from 59-65 for the low sensitivities, 75-81 for medium and 85-88 for high. Summary statistics of the median intensity of arrays at low, medium, and high scan (Table 1) revealed that the low scan in the present study had an average normalized log2 median intensity of 7.27 and ranged from 7.24 to 7.28. In comparison, the medium sensitivity yielded a median signal intensity of 8.95 and ranged from 8.91 to 8.98. The high scan ranged from 9.86 to 9.97 with a median of 9.91 across the 40 microarrays.

**Table 1:** Summary statistics of the median intensity of arrays at low, medium, and high scan.

Scan	Mean	Median	Standard Deviation	Minimum	Maximum
Low	7.26	7.27	0.01	7.24	7.28
Medium	8.95	8.95	0.02	8.91	8.98
High	9.91	9.91	0.02	9.86	9.97

**Figure 1:**
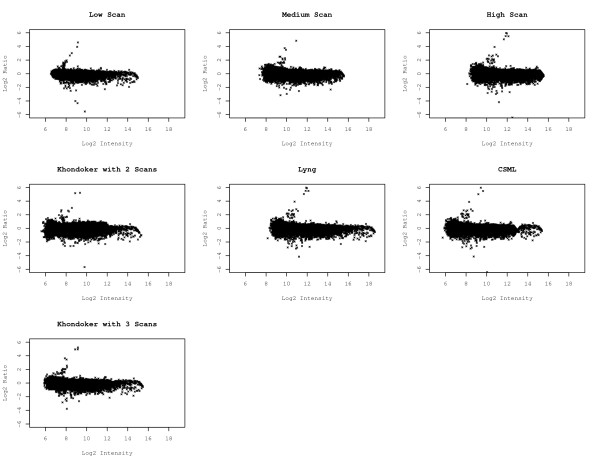
**Ratio intensity plots using cyclic lowess normalized data**.

To examine the impact of scanning intensity and multiple scan approaches on the dataset as a whole, ratio-intensity plots were generated for all individual and multiple scan approaches using the cyclic lowess normalized data (Figure 1). The range of average intensities for the individual scans was 7-8 units. Increasing variation in the log ratio was observed with increasing PMT. This was especially seen in the lower range (i.e. in the background). The log2 intensity range appeared somewhat compressed at higher PMT. We used data captured from multiple scans of each array to compare the following three methods: (1) a method by Lyng et al. [1], which estimates a multiplicative scaling effect using a subset of probes with high signal intensities not influenced by saturation; (2) the clipping saturation maximum likelihood approach (CSML) from García de la Nava et al. [2], which estimates the multiplicative scaling effect using all probes by employing a robust regression approach; and (3) the Khondoker et al. [3] method using the multiscan library [15] in R [16], which employs a functional regression model assuming the expression data is Cauchy distributed. This latter method allows incorporation of any number of scans, and so it was assessed using both 2- and 3-scan cases; in contrast, the first two methods are limited to using data from two scans (low and high). The three multiple scan approaches yielded an average intensity range of roughly 10 units, although the data acquired using the approach of Lyng et al. was shifted by two units (8-18) compared to the other two methods (6-16). Of the multiple scan approaches, the 3-scan case of Khondoker et al. appeared to have the least amount of variation in relative intensity, and was comparable to the low scan. In contrast, the 2-scan case of the Khondoker et al. method appeared to have considerable variability, particularly among low intensity probes. For the García de la Nava et al. CSML method, the log ratio was observed to approach 0 at about 13 units. This was not observed for the individual scans or for either of the two other multiple scan approaches.

**Figure 2:**
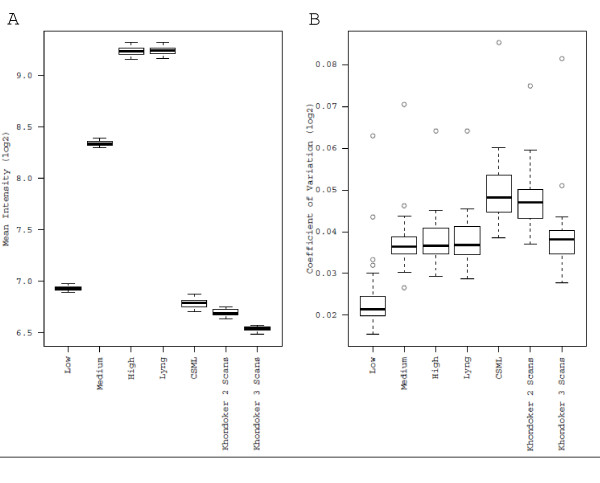
**Mean and coefficient of variation for the negative control probes**. A) Comparative boxplots of the mean of the 162 negative control probes for each of the 40 microarrays were generated from the cyclic lowess normalized data. B) Comparative boxplots of the coefficient of variation of the 162 negative control probes for each of the 40 microarrays were generated from the cyclic lowess normalized data.

To examine how each approach affected the background fluorescence, the mean fluorescence and coefficient of variation of negative control probes were measured. For the individual scans, higher background was observed with increasing PMT (Figure 2a). Among the approaches that synthesize data across multiple scans, negative control means were roughly equal between the Lyng et al. method and the high scan. In contrast, CSML and the Khondoker et al. method using 2 or 3 scans exhibited considerably lower mean values, equal to or less than the low scan. The inter-quartile ranges were similar across all scans and methods, suggesting high reproducibility of the negative controls across microarrays. Comparison of the coefficient of variation of the negative control spots for individual scans revealed that the low scan had the lowest value (Figure 2b). While the average coefficients of variation for the medium and high scans were similar, the inter-quartile range for the high scan was considerably larger. All multiple scan approaches displayed coefficients of variation that were greater than the low scan. The CSML and Khondoker et al. 2-scan case exhibited the highest coefficient of variation, and the CSML method had the most variable coefficient of variation.

**Figure 3:**
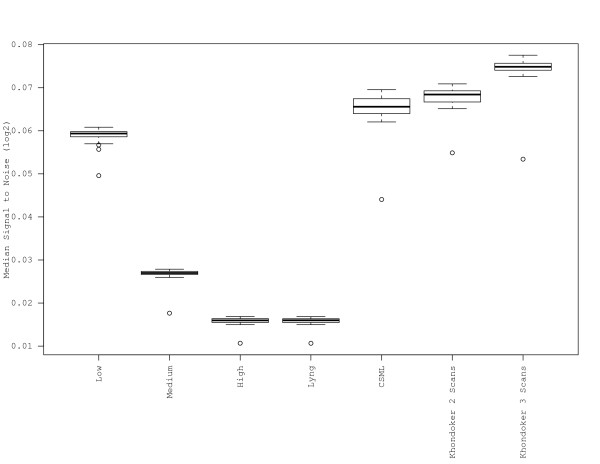
**Signal-to-noise ratio**. The median of the normalized data (log2) minus the mean of the negative controls for each of the 40 microarrays was used to generate comparative boxplots for each of the individual scans and the three multiple scan approaches.

Since probe detection is dependent on signal strength in addition to the level of background florescence, we examined the SNR for all scans and for the multiple scan approaches. Here the SNR was defined as the median of the log2 of the foreground signal intensity divided by the mean of the negative controls. SNRs for the individual scans decreased with increasing scanner intensity (Figure 3). Of the multiple scan approaches, the SNRs for the Khondoker et al. (2- and 3-scan) and CSML methods were highest, with mean values greater than the low scan. The SNR determined for the Lyng et al. method was the lowest among multiple scan approaches, and was identical to that of the high scan. Note that Figure 3 is virtually the inverse of Figure 2a, implying that the background is the defining component of the SNR.

**Figure 4:**
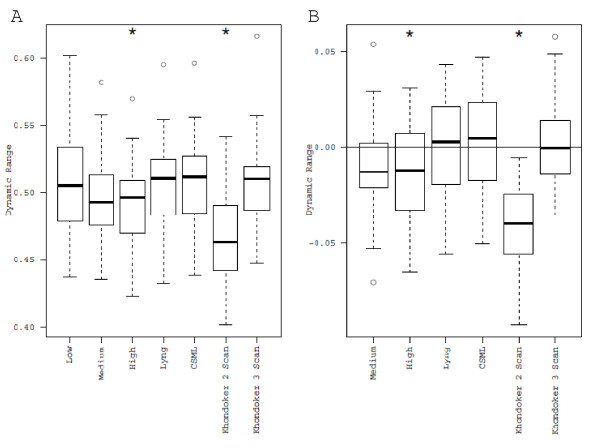
**Box-plots of the dynamic range for individual and multiple scan approaches**. A) Proportion of useable data (probes above background and below 10% of saturation) plotted for all approaches. B) Difference in proportion of useable data for each approach relative to the low scan (indicated by the horizontal line). * indicates significantly different from the low scan, paired t-test, p < 0.05 after Bonferroni correction).

To investigate whether multiple scan techniques increase the dynamic range, we generated box plots of the percentage of useable data (i.e. data above the mean plus three standard deviations of the negative control probes and less than 10% of saturation) for the 40 arrays (Figure 4a). Comparison of the individual scans revealed that the dynamic range of the low scan was significantly greater than that of the high scan (paired t-test, Bonferroni-adjusted p = 0.0144), but was not significantly different from the medium scan (paired t-test, Bonferroni-adjusted p = 0.1047). The Khondoker et al. method with 2 scans had the lowest percentage of useable data, and was statistically different compared to all other approaches (paired t-test, Bonferroni-adjusted p < 0.05). Since it is hypothesized that multiple scan approaches increase the dynamic range relative to individual scans, we compared all other approaches to the low scan, as this scan yielded the greatest dynamic range of the individual scans (Figure 4b). There was no significant improvement of any multiple scan approach relative to the low scan. Other significant differences (Lyng > high; CSML > medium, high; Khondoker > medium, high; CSML > Lyng) are presented in Additional File 1: Table S1.

**Figure 5:**
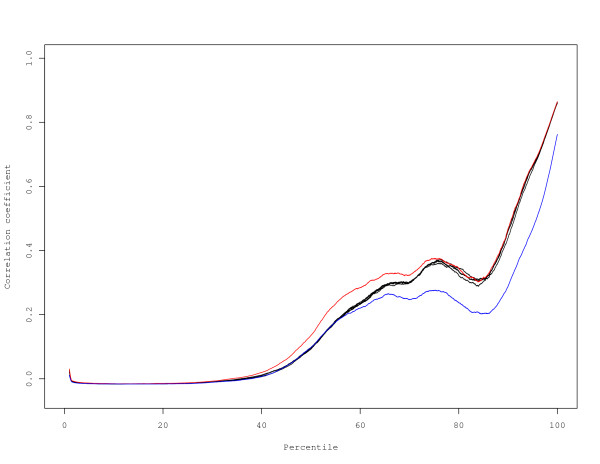
**Correlation by intensity**. A sliding window of 2000 probes was used to estimate the correlation from the low intensity to the high intensity probes. All correlations were aligned by percentile to plot the correlation by intensity for the individual scans and multiple scan methods. Coloured lines indicate methods that display significant differences from all other methods over some interval. Blue, the Khondoker et al. method with 2 scans. Red, the Khondoker et al. method with 3 scans.

In order to compare the reproducibility of results obtained using individual scans at different sensitivities (low, medium, high) or using a multiple scan approach, we performed a Spearman correlation analysis on 40 technical replicates using the entire dataset of 22575 probes. This analysis revealed very little difference among the various approaches, with correlations ranging from 0.8586 to 0.8812 (Table 2). The only approach that was statistically significant after Bonferroni correction was the Khondoker et al. approach using 3 scans, which yielded a slightly higher correlation coefficient than the other approaches. Given the similar reproducibility for all approaches when using the entire dataset for calculation of the Spearman correlation, we examined whether correlations varied across the intensity spectrum. Stratification of all 22575 probes into percentiles revealed that while most approaches yielded remarkably similar correlation estimates across the intensity range, the Khondoker et al. 2-scan and 3-scan results deviated from the other approaches (Figure 5). All scans and methods had poor correlation for the lower percentiles, and correlations improved as probe intensity increased. In order to statistically test for differences among the approaches, we stratified probe intensities by decile, and performed Spearman correlations (Additional File 2: Table S2). Comparison of individual scans revealed no significant improvement in the reproducibility of low intensity probes at higher PMT settings. Remarkably, the Khondoker et al. 2-scan case resulted in lower correlations over the range 70^th ^to 100^th ^percentile. In sharp contrast, the 3-scan case yielded a statistically significant improvement in reproducibility over the 30^th ^to 60^th ^percentile. There were no other significant differences among the approaches. Our data indicate that scanning at higher PMT settings does not result in any gain in reproducibility for low intensity probes. Indeed, the low scan yielded equivalent correlation estimates to the medium and high scans across the entire intensity range. Although correlations were generally poor for probes in the background, our data indicate that use of a multiple scan approach can improve somewhat the reproducibility of these probes.

**Table 2:** Spearman correlations for all scans and methods using the entire dataset.

		Bonferroni Adjusted 95% CI	
		
Scan	Mean	Lower Limit	Upper Limit
Khondoker with 3 Scans	0.8812	0.8729	0.8858
CSML	0.8670	0.8602	0.8727
High	0.8658	0.8599	0.8717
Lyng	0.8657	0.8595	0.8712
Low	0.8643	0.8569	0.8705
Medium	0.8636	0.8569	0.8706
Khondoker with 2 Scans	0.8586	0.8524	0.8637

The mean and 95% Bonferroni-adjusted confidence intervals estimated using the bootstrap are displayed for each dataset.

**Figure 6:**
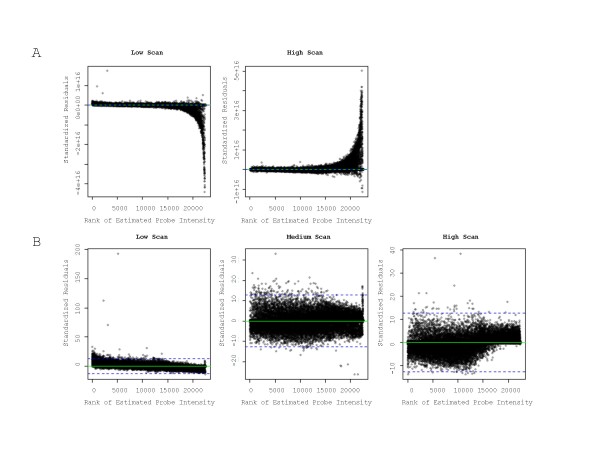
**Residual plots of the Khondoker et al. 2- and 3-scan cases**. Standardized residuals were plotted against rank of estimated probe intensity. A) 2-scan case. B) 3-scan case.

To investigate the substantial difference in reproducibility between the 2- and 3-scan cases of the Khondoker et al. method, we generated residual plots of the model fit. We observed clear differences in the residual plots for the two cases (Figure 6). For the 2-scan case, there was a significant lack-of-fit for probes in the high intensity range (Figure 6a). In contrast, in the 3-scan case there was a better fit for data across the entire intensity range (Figure 6b). To verify that the lack-of-fit was not specific to this dataset, we examined the example (4 scans of a single array for 1000 probes) provided in the multiscan R library [15] associated with this method. Using these data, we applied the Khondoker et al. method for the 2- and 3-scan cases. For the 2-scan case (i.e. using only low and high scans) we observed a similar lack-of-fit, whereas for the 3-scan case no significant lack-of-fit was observed (data not shown), consistent with the results presented here. Khondoker et al. [3] state that for functional regression models there are problems when estimating separate scaling terms for each beta coefficient as the likelihood function goes to infinity if any one of the variance parameters goes to zero. As a result, their model assumes that the scale parameters increase in proportion to the beta coefficients across scans. For our data, and for the data provided with the multiscan R library [15], at least one of the σ_1 _and σ_2 _terms approaches 0 for the 2-scan case (data not shown). This was not apparent when 3 or more scans were used, and may explain the difference in reproducibility between the 2- and 3-scan cases.

**Figure 7:**
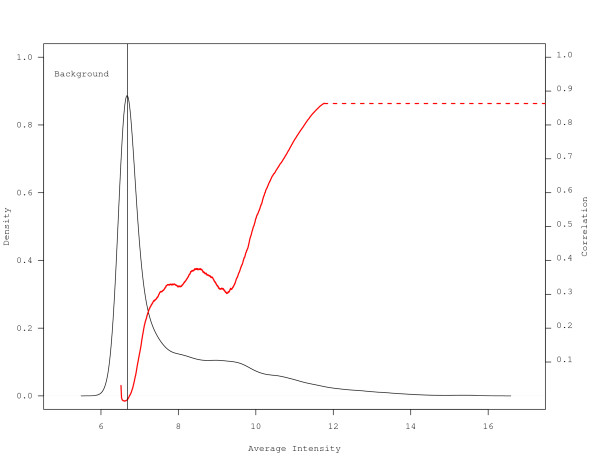
**Distribution of average probe intensity**. A data display of the density of probe intensity was generated using the Khondoker et al. method for 3 scans. The correlation based on the average intensity for the probes within the sliding window was overlaid in red. The vertical line indicates the upper bound of the background estimated by the mean plus three standard deviations of the negative controls. The red dashed line extends the correlation over the range of the data to the second y-axis.

To investigate the poor correlation observed for the lower deciles, the average distribution of probe signal intensities was plotted with overlaying correlation estimated using a sliding window of 2000 probes for each approach. As the Khondoker et al. 3-scan case had the highest correlation estimates, it is presented in Figure 7. Plots for all other approaches are presented in Additional File 3: Figure S1. For all approaches, approximately 50% of the data were judged to be within the background as identified by the vertical line at the mean plus three standard deviations of the negative control probes. Correlation estimates were poor for all datasets below the 50^th ^percentile, and improved as the average intensity increased beyond the 50^th ^percentile.

## Discussion

Applying optimal scanner settings for microarray experiments greatly enhances data acquisition. However, defining what is optimal is a challenge, and there is little consensus on how to achieve an optimal scan. Specific average intensities have been put forth as guidelines [4,12], but actual PMT settings must be determined empirically. Some argue that scanner settings should be set to maximize the SNR [1]. Others argue that one should minimize censoring due to saturation [3]. Yang and Speed [17] found that after normalization, the ranks or ordering for the majority of genes remained the same at different PMTs, indicating that for most probes PMT setting may be irrelevant. Combining scans at different intensities has been suggested as an approach to yield improved datasets [1-10]. In the present work we objectively compared individual scans at three different intensities, and evaluated the utility of three algorithms that incorporate data from multiple scans using several metrics for assessing data quality.

Sharov et al. [12] suggested that the sensitivity placing the greatest amount of data within the limits of the quantifiable range is approximately 8 FLUs (log2). However, it was argued that this may be too close to background, and so an average value between 10 and 12 was suggested as a good balance between having too many probes with intensities that are close to background vs. too many approaching saturation [12]. The low scan used in the present work, with a FLU of ranging from 7.24 to 7.28, was therefore well below the suggested optimal level, the medium scan (8.91-8.98) was close to the "optimal sensitivity", while the high scan (9.86-9.97) approached the "well-balanced" level (Table 1). Our choice of scanner sensitivities therefore spans a relevant range of PMT settings.

Changes to the scanner sensitivity and the act of combining data generated from multiple scans will alter the distribution of signal intensities. Examination of ratio-intensity plots revealed extension of the range in the average intensity for the multiple scans compared with the individual scans (Figure 1). However, it is unclear whether the increased range is meaningful in terms of improved data quality. Indeed, in the present study there was no clear relationship between increased intensity range and improved reproducibility. Further examination of ratio-intensity plots revealed additional differences among the multiple scan approaches examined. The clipping saturation model used here, which incorporates a maximum likelihood approach, was previously shown to provide a smooth transition for various datasets [2]. In contrast, pinching of data was observed using the threshold approach for the gamma saturation model [2]. Our data show that a similar artifact can also be observed using the maximum likelihood approach. Such artifacts raise concern regarding possible distortion of the data using this method. It is also noteworthy that the 2-scan and 3-scan cases of the Khondoker et al. method yielded quite different ratio-intensity plots, with the 2-scan case exhibiting considerably greater variability than the 3-scan case. The differences in the ratio-intensity plots suggest that visual displays of the data are useful for rapid examination of data quality.

While scanning at higher sensitivities has been proposed as an approach to improve quantification of low intensity probes [1,8,11], there may be a corresponding increase in the background noise, which would impact the SNR. In the present study, the mean and coefficient of variation of the negative controls were both significantly higher for the medium and high scans compared to the low scan (Figure 2), confirming an increase in the background noise at higher PMT settings. However, this might not be considered relevant if probe signal intensities increase at a greater rate than the background noise with increased scanning sensitivity, since this would result in an improved SNR. Remarkably, investigation of SNRs for the three scanning sensitivities and the multiple scan approaches revealed that the low scan had a median SNR that was considerably higher than that for the medium and high scans (Figure 3). Therefore, our data indicate that maximizing the SNR may not be achieved by scanning at higher PMT setting, since any gains achieved by increasing the PMT appear to be lost due to the increase in background signal. Rather, our data suggest that using this metric, a low scan may be more appropriate. A further advantage of the low scan is that it avoids the need to censor a large proportion of probes due to signal saturation, as discussed previously [3]. There was considerable disparity among the multiple scan approaches used with respect to mean and coefficient of variation of the negative controls and the median SNR. The relative success of the Khondoker et al. and CSML approaches in generating the highest SNRs likely relates to the lower mean of the negative control probes. The Lyng method replaces values from the high scan that are above a certain threshold (15.6FLUs) with data from the low scan, adjusted by a correction factor. Thus, the high background observed using the Lyng approach is not unexpected as the method is aimed towards addressing censoring due to saturation and not to maximize SNR.

Incorporation of multiple scans should theoretically enable one to obtain more useable data by reducing the impact of quantification error and censoring due to saturation. Comparison of the three multiple scan approaches against the individual scan with the greatest dynamic range (the low scan) revealed no significant improvement in dynamic range (Figure 4). We have defined dynamic range as the proportion of probes that have signals above background (mean plus three standard deviations of the negative control probes) and below 10% of saturation. For multiple scan approaches, the upper end of the dynamic range is determined by the low scan. To increase the dynamic range relative to the low scan would therefore require a reduction in the estimated background. The mean of the negative control probes was indeed reduced by applying specific multiple scan approaches (CSML, Khondoker et al. 2-scan and 3-scan cases; Figure 2a). However, the coefficient of variation for these probes was considerably higher compared to the low scan when a multiple scan approach was applied (Figure 4b). According to these data, the higher coefficient of variation observed for the multiple scan approaches appears to be responsible for the lack of improvement in dynamic range compared to the low scan. This may be a result of lack-of-fit of the statistical models employed for probes in the background. If multiple scan approaches are to increase the dynamic range, there would have to be improvements in how the approaches handle data for low intensity probes.

In comparing multiple scan approaches, impacts on reproducibility should be considered, as increased reproducibility should translate into improved ability to detect differential gene expression. Comparison of correlation estimates based on the dataset as a whole revealed little difference among the various approaches, with only the 3-scan case of the Khondoker et al. approach yielding a statistically higher correlation (Table 2). However, it is recognized that low abundance genes exhibit lower reproducibility while genes with higher intensity tend to have improved reproducibility [18]. This implies that reproducibility differs across the entire distribution of signal intensities. We therefore partitioned the distribution of signal intensities into percentile bins, thereby improving the resolution in order to detect differences among the approaches. The analysis revealed that over specific intensity ranges, approaches differed with respect to data reproducibility (Figure 5). There was no indication from our data that incorporation of a multiple scanning approach improved data quality below the 30^th ^percentile. It is important to note that roughly 50% of probes were deemed to be within background (Figure 7 and Supplemental Figure 1). Also, there were no gains in reproducibility for probes greater than the 70^th ^percentile. However, for probes within the 30^th ^to 60^th ^percentile, the Khondoker et al. 3-scan case yielded statistically higher correlations compared to all individual scans and multiple scan approaches (Additional File: Table S2). Remarkably, the 2-scan case yielded statistically lower correlations compared to all other approaches, including single scans, indicating that the number of scans is a critical factor for data reproducibility using this method. The residuals for high intensity probes indicate that the assumptions for the model may not have been satisfied in the 2-scan case (Figure 6a). Our data indicate that the correlation estimates are driven primarily by the high intensity probes (Additional File 2: Table S2), and so the increased variance for these probes in the 2-scan case may result in the lower reproducibility observed. The data reveal that 1) not all multiple scan approaches are equal; 2) multiple scan approaches can yield more reproducible data than individual scans; and 3) the number of scans used can markedly impact data quality, including reducing the quality of the data below levels seen for individual scans.

Overall the data show that incorporation of either of the CSML or the Khondoker et al. multiple scan approaches yielded high SNR, low variability of negative controls, extended probe intensity ranges, and high correlations among samples. Given the improved reproducibility of data that can be achieved, it may be desirable to use a multiple scan approach despite the additional scanner and computational time required. A further advantage of using a multiple scan approach is that "optimal" PMT settings need not be estimated in advance or determined empirically, as the user simply scans at a number of PMT settings and applies a multiple scan approach. Issues associated with how well the data fit the model are a concern, as shown here using the CSML and Khondoker et al. 2-scan case. Most importantly, a significant concern is the possibility that application of a multiple scan approach could actually reduce data quality, as was observed for the Khondoker et al. 2-scan case. Assessment of data quality using ratio-intensity plots or other metrics is advisable.

## Conclusions

In summary, using data from a study involving a large number of technical replicates enabled comparison of single scan and multiple scan approaches. Our results revealed clear differences in the distribution of the data, background, SNR, and reproducibility of the data among the methods. The algorithm developed by Khondoker et al. with 3 scans produced the best data according to the metrics assessed in this study. A further advantage of the Khondoker et al. approach is that it can accommodate any number of scans, unlike the Garcia de la Nava and Lyng approaches, which use two scans only. However, our data suggest that the Khondoker et al. approach may not be suitable for use with fewer than 3 scans. While incorporation of a multiple scan approach may not be warranted in all situations, it has the potential to yield better quality and more reproducible data than any individual scan.

## Methods

### Sample preparation, hybridization, and scanning

The datasets used here comprise 40 technical replicates and are publicly available in the NCBI Gene Expression Omnibus database (Accession # GSE19493; http://www.ncbi.nlm.nih.gov/projects/geo). Universal Mouse Reference RNA (Stratagene, CA, USA) was labelled with Cy3 and hybridized to Agilent Mouse G4121A Microarrays. The Cy5 channel measured lung mRNA levels from a toxicology experiment described elsewhere [14]; these were not analysed for the present study. Arrays were incubated overnight at 60°C in Agilent hybridization solution and washed according to manufacturer's instructions. Arrays were scanned at three different PMT settings (Low: 59-65, Medium 75-81, High 85-88) using a ScanArray Express (Perkin-Elmer Life Sciences, Woodbridge, ON, Canada), and data were acquired with ImaGene 5.5 (BioDiscovery, CA, USA).

### Processing and normalization

Non-background subtracted median signal intensity data from the low and high scans were read into R 2.9.0 [16]. Background was estimated as the mean plus 3 standard deviations of the (-)3xSLv1 negative control probes. For the Lyng et al. method, probes with signal intensities between 20,000 to 30,000 FLUs in the high scan were identified. For each of these probes the ratios of the high scan signal intensity to the low scan signal intensity were calculated. The average of these ratios provided the correction factor that was multiplied with the low scan data to impute the corresponding values in the high scan above 50,000 FLUs. For implementing the CSML method, a scatterplot of the low scan versus the high scan sensitivity was constructed to identify the type of saturation (clipping saturation or gamma saturation). Our data exhibited clipping saturation (data not shown). The clipping saturation maximum likelihood approach was written in R. Here the lqs function in the MASS library [19] was used to robustly estimate the required parameters. The Khondoker et al. method was applied to the low, medium, and high scans using the multiscan R library [15]. All data from individual scans and resulting data from the methods using multiple scans were normalized using the cyclic lowess [20] approach in R using the normalize.loess function in the affy library [21].

### Dynamic range analysis

For individual scans, dynamic range was defined as the proportion of data that had signal intensities greater than background and less than 10% of saturation. For multiple scans, the dynamic range was defined as proportion of data that had signal intensities greater than background and less than 10% of saturation of the low scan. Paired t-tests were conducted to identify differences among between all approaches. The p-values were adjusted for multiple comparisons using the Bonferroni approach.

### Correlation analysis

In order to ascertain the reproducibility of the technical replicates, a correlation analysis was done following the methodology outlined in [22]. Rank transformation of the normalized data was used to estimate the correlation matrix. The bootstrap was applied to obtain Bonferroni-adjusted 95% percentile confidence intervals (B = 10,000) for the average correlation for each of the individual scans and for the three multiple scan methods. The correlation analysis was also conducted by stratifying the intensity range of the probes into percentiles based on the median signal intensity for the 40 arrays. For each of the percentiles the estimated correlation and 95% Bonferroni-adjusted confidence intervals (B = 10,000) were obtained.

## Authors' contributions

AW and EMT conceived of the study, planned the approaches used, analysed the data, and wrote the manuscript. EMT performed the microarray hybridizations and data capture. AW carried out the data processing and analysis. AW and EMT have read and approved the final manuscript.

## Supplementary Material

Additional file 1 **Pairwise comparisons of the proportion of useable data for all approaches**. Paired t-tests were conducted for all possible pairwise combinations to identify differences in the proportion of useable data. The estimated difference, p-value and Bonferroni adjusted p-value for each comparison are presented. Click here for file

Additional file 2 **Spearman correlations by intensity using percentile bins**. For each of the percentiles determined from the average intensity distribution, the mean and 95% bootstrap percentile confidence intervals are displayed in brackets. Highlighted results indicate significant differences by comparing 95% Bonferroni adjusted confidence intervals for all scans and methods. Orange, significantly higher correlations compared to all other methods. Blue, significantly lower correlations compared to all other methods. Click here for file

Additional file 3 **Distribution of average probe intensity**. The distribution of probe intensity was generated for each single scan and multiple scan method. Overlaid in red is the correlation based on the average intensity for the probes within the sliding window. The upper bound of the estimated background is indicated by the vertical line. The red dashed line extends the correlation over the range of the data to the second y-axis. Click here for file
