# Supplementary figures and images for: Effects of scanning sensitivity and multiple scan algorithms on microarray data quality
Source: BMC Bioinformatics. 2010 Mar 12;11:127. doi: 10.1186/1471-2105-11-127 (PMC2846908; doi:10.1186/1471-2105-11-127)

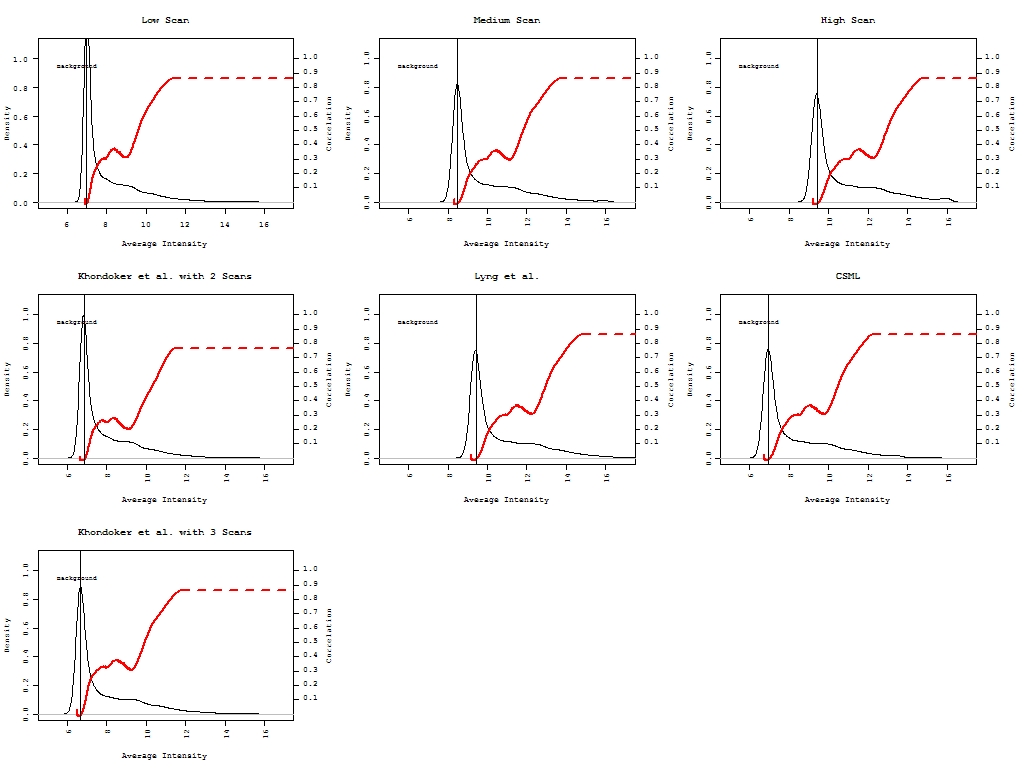

Supplement: Additional file 3 — Distribution of average probe intensity. The distribution of probe intensity was generated for each single scan and multiple scan method. Overlaid in red is the correlation based on the average intensity for the probes within the sliding window. The upper bound of the estimated background is indicated by the vertical line. The red dashed line extends the correlation over the range of the data to the second y-axis. [file 1471-2105-11-127-S3.JPEG]
